# Effects of temporal and spatial scales on soil yeast communities in the peach orchard

**DOI:** 10.3389/fmicb.2023.1226142

**Published:** 2023-09-19

**Authors:** ShanShan Zhu, YanLi Cai, Yang Li, Jie Xiong, YongHui Lei, YanFei Sun

**Affiliations:** ^1^Department of Plant Protection, College of Agriculture, Shihezi University, Shihezi, Xinjiang, China; ^2^College of Life Sciences, Shihezi University, Shihezi, Xinjiang, China

**Keywords:** yeast community, spatio-temporal scales, high-throughput sequencing, co-occurrence network, orchard soil

## Abstract

Shihezi Reclamation Area is located at the southern edge of the Junggar Basin, with natural, soil, and climatic conditions unique to the production of peaches. In turn, peach orchards have accumulated rich microbial resources. As an important taxon of soil fungi, the diversity and community structure changes of yeast in the soil of peach orchards on spatial and temporal scales are still unknown. Here, we aimed to investigate the changes in yeast diversity and community structure in non-rhizosphere and rhizosphere soils of peach trees of different ages in the peach orchard and the factors affecting them, as well as the changes in the yeast co-occurrence network in the peach orchard at spatial and temporal scales. High-through put sequencing results showed that a total of 114 yeast genera were detected in all soil samples, belonging to Ascomycota (60 genera) and Basidiomycota (54 genera). The most dominant genus, *Cryptococcus*, was present in greater than 10% abundance in each sample. Overall, the differences in yeast diversity between non-rhizosphere and rhizosphere soil of peach trees at 3, 8 and 15 years were not significant. Principal coordinate analysis (PCoA) showed that differences in yeast community structure were more pronounced at the temporal scale compared to the spatial scale. The results of soil physical and chemical analysis showed that the 15-year-old peach rhizosphere soil had the lowest pH, while the OM, TN, and TP contents increased significantly. Redundancy analysis showed that soil pH and CO were key factors contributing to changes in soil yeast community structure in the peach orchard at both spatial and temporal scales. The results of co-occurrence network analysis showed that the peach orchard soil yeast network showed synergistic effects as a whole, and the degree of interactions and connection tightness of the 15-year-old peach orchard soil yeast network were significantly higher than the 3- and 8-year-old ones on the time scale. The results reveal the distribution pattern and mechanism of action of yeast communities in peach orchard soils, which can help to develop effective soil management strategies and improve the stability of soil microecology, thus promoting crop growth.

## 1. Introduction

Yeast is known as the first “domesticated microorganism” in human history and is widely used in food fermentation, industry and agriculture, and pharmaceutical production because of its short growth cycle, high metabolic efficiency, and production of beneficial metabolites (Zhao et al., 2004). In recent years, yeasts have been shown to play a key role also in the biosorption of heavy metal ions in the environment, in building the balance of ecosystems, and in the prevention and control of polluted environments (He et al., 2022; Igwegbe et al., 2022). Yeast has an extremely wide distribution habitat, and since it prefers acidic and sugar-rich habitats, it is usually found in orchards, mainly from different organs of fruit trees such as leaves, flowers and fruits, as well as from orchard soil (Yang and Wang, 2009). Not only can novel yeast species be found in the orchard soil (Chen et al., 2010, 2012), but also many yeasts with excellent performance can be screened to add unique flavor to the fruit wine (Chen et al., 2018; Wang et al., 2022). There are also some orchard soil yeasts that can be used to control fruit tree diseases or to alleviate post-harvest diseases of fruits to extend the shelf life of fruit (Wang et al., 2016; Ferraz et al., 2019). In addition, some soil yeasts can indirectly promote plant root growth and development by enhancing colonization of arbuscular mycorrhizal (AM) fungal in host plants (Boby et al., 2008; Mirabal Alonso et al., 2008), and there are also yeasts that possess the ability to solubilize rock phosphate or produce plant growth regulators to improve plant growth directly (Nassar et al., 2005; El-Tarabily and Sivasithamparam, 2006; Kumla et al., 2020). Because of this, soil conditioners containing yeast have been developed to improve crop productivity (Ito and Ito, 2001). Research is also ongoing on the potential of soil yeast as a biofertilizer, which can also reduce the damage to the soil caused by conventional chemical fertilizers to some extent and rebuild the maintenance capacity of agroecosystems (Hernández-Fernández et al., 2021; Marques et al., 2021). Therefore, the study of yeast diversity in orchard soils is still of great importance and can provide rich strain resources for social production. In fact, in addition to exploring the function of soil yeasts in orchards, the search for relevant factors affecting the diversity and composition of yeast communities has also never stopped.

Soil yeast diversity and community structure are generally influenced by factors such as soil type, type of vegetation covered, climatic conditions, and geographic location (Vadkertiová et al., 2017). For instance, in vineyards, different grape types’ cultivated soils varied in their diversity of yeast composition and abundance of the same yeast species, and there was a significant relationship between some yeast species and particular grape varieties (Zhang et al., 2017). Moreover, distinct yeast strains obtained from various vineyard soils resulted in noticeably diverse fermentation flavors, providing directions for future screening of edible grape yeasts and genetic improvement of edible grapes (Wang et al., 2022). Yeast populations living in soil under various fruit tree species varied in species richness and evenness, with the highest species richness in soil next to apricot trees, according to a previous study of yeasts in 200 soil samples from five fruit tree species (apple, pear, plum, peach, and apricot) in two regions of Slovakia (Vadkertiová et al., 2019). Numerous earlier studies have demonstrated that the number of yeast cells tends to decrease as soil depth increases. This is because deeper soil contains less efficient nutrients and soil organic matter, which is unfavorable for the survival of yeast (Starmer and Lachance, 2011; Yurkov, 2018). As well, rhizosphere soil yeasts are more numerous than bulk soil due to their ease of uptake of simple organic carbon compounds secreted by plant roots and their ability to feed on spoilage fruits deposited in the soil (Cloete et al., 2009; Botha, 2011). Understanding the survival mechanism of yeast in orchard soil ecosystem is crucial for the growth and application of yeast resources.

Peach is rich in many essential substances for human body, including protein, crude fiber, various amino acids, carotenoids and minerals such as iron and phosphorus (Yin et al., 2017), and is known as the “first fruit of the world” (Zhou and Zhang, 2009). Shihezi, Xinjiang, is located in the middle of the northern foot of Tianshan Mountains and the southern edge of Junggar Basin (Fan et al., 2006). It has a dry climate, abundant light, heat and water sources, a wide day-night temperature range, long daylight hours, drought, and little rain during the fruiting season of fruit trees, all of which are very conducive to the accumulation of sugar and dry matter in fruits as well as the accumulation of rich yeast resources (Xu et al., 2015). Previous studies on microorganisms in peach orchards have focused either on the biological control of postharvest peach fruit diseases, on the structural analysis of peach leaf-attached yeast communities in peach orchards by a culture-independent method, or on the culturable yeast diversity of soil in peach orchards by a culture-dependent method (Yin et al., 2017; Liu et al., 2019; Wang et al., 2019). However, the yeast diversity and community composition in non-rhizosphere and rhizosphere soils of perennial peach trees containing both culturable and nonculturable yeasts, and whether they are influenced by annual (yearly) variation and soil factors, are not yet known, which will be a bottleneck for studying yeast adaptation mechanisms in peach orchards and their further development and utilization.

As a natural breeding ground for microorganisms, each gram of soil may contain millions of microbial species (Bunge et al., 2006), and most (>99%) are non-culturable microorganisms. Illumina MiSeq high-throughput sequencing technology allows for more comprehensive and accurate detection of species composition compared to traditional culture-dependent methods (Zebin et al., 2016). Here, we studied the diversity and composition of yeast communities associated with the non-rhizosphere and rhizosphere soil of peach trees of different ages based on Illumina MiSeq high-throughput sequencing technology in peach orchard of Shihezi, Xinjiang. The aim was to explore the differences in soil yeast diversity and community structure in the peach orchard at temporal and spatial scales, the correlation between soil factors and yeast communities, the variation of the soil yeast co-occurrence network in the peach orchard at spatial and temporal scales, and the mechanism of yeast action in the network. Our study provides supplemental information for a comprehensive understanding of peach orchard yeast resources, factors affecting soil yeast composition in perennial peach trees, and the mechanisms of action among yeasts in peach orchard soils, as well as some ideas for achieving sustainable agricultural development in the peach orchard.

## 2. Materials and methods

### 2.1. Study site and sample collection

Our soil samples were collected from a peach orchard in Shihezi 143rd Regiment (N44°28′, E85°82′, Altitude 450 m), Xinjiang. The soil type is gray desert soil with a medium loamy texture, cultivated at a depth of 80–100 cm with good permeability, and the peach trees are planted with a spacing of 5.0 m between plants and 6.0 m between rows, with a density of 330 plants per hectare (ha). The site is planted on 500 hectares and has year-round good irrigation conditions. All peach trees of the peach orchard are *Amygdalus persica* L. “Compressa.” The climate of the Shihezi region is typical of a temperate continental climate, with long and severe winters and short and hot summers (Han et al., 2008). The climate information of the sampling sites is shown in Table 1. The data of precipitation (PRECTP) and temperature (TEMP) from NOAA—Climate Prediction Center,^1^ land surface temperature (LST) and relative humidity (RH) from NASA GES DISC MERRA2—inst1_2d_asm_Nx,^2^ evaporation land (EVLAND) from NASA GES DISC MERRA2—tavg1_2d_lnd_Nx (see footnote 2).

**Table 1:** Meteorological data of Shihezi region in the past 10 years.

Year	PRECTP (mm)	TEMP (°C)	LTS (°C)	RH (%)	EVLAND (mm)
2013	18.15 ± 3.743	8.00 ± 2.993	7.08 ± 3.448	48.14 ± 1.463	24.95 ± 4.948
2014	16.19 ± 2.993	6.65 ± 3.320	5.85 ± 3.706	46.33 ± 2.519	22.99 ± 4.833
2015	21.86 ± 3.808	7.81 ± 3.071	6.96 ± 3.546	49.15 ± 2.225	26.20 ± 5.266
2016	22.94 ± 4.876	7.81 ± 3.149	7.00 ± 3.600	52.67 ± 1.970	30.89 ± 6.938
2017	16.83 ± 3.469	7.56 ± 3.152	6.81 ± 3.548	49.31 ± 2.436	24.87 ± 5.440
2018	17.85 ± 3.003	6.29 ± 3.499	5.25 ± 4.025	50.57 ± 2.506	23.02 ± 4.079
2019	17.62 ± 4.083	7.28 ± 3.245	6.28 ± 3.714	50.32 ± 2.436	23.54 ± 4.746
2020	16.45 ± 3.415	7.09 ± 3.143	6.16 ± 3.628	48.30 ± 2.116	24.01 ± 4.651
2021	15.97 ± 2.746	7.54 ± 3.136	6.53 ± 3.611	46.47 ± 2.082	22.56 ± 4.193
2022	13.50 ± 2.042	8.02 ± 3.375	7.26 ± 3.870	44.46 ± 2.731	16.43 ± 3.027

**Figure 1:**
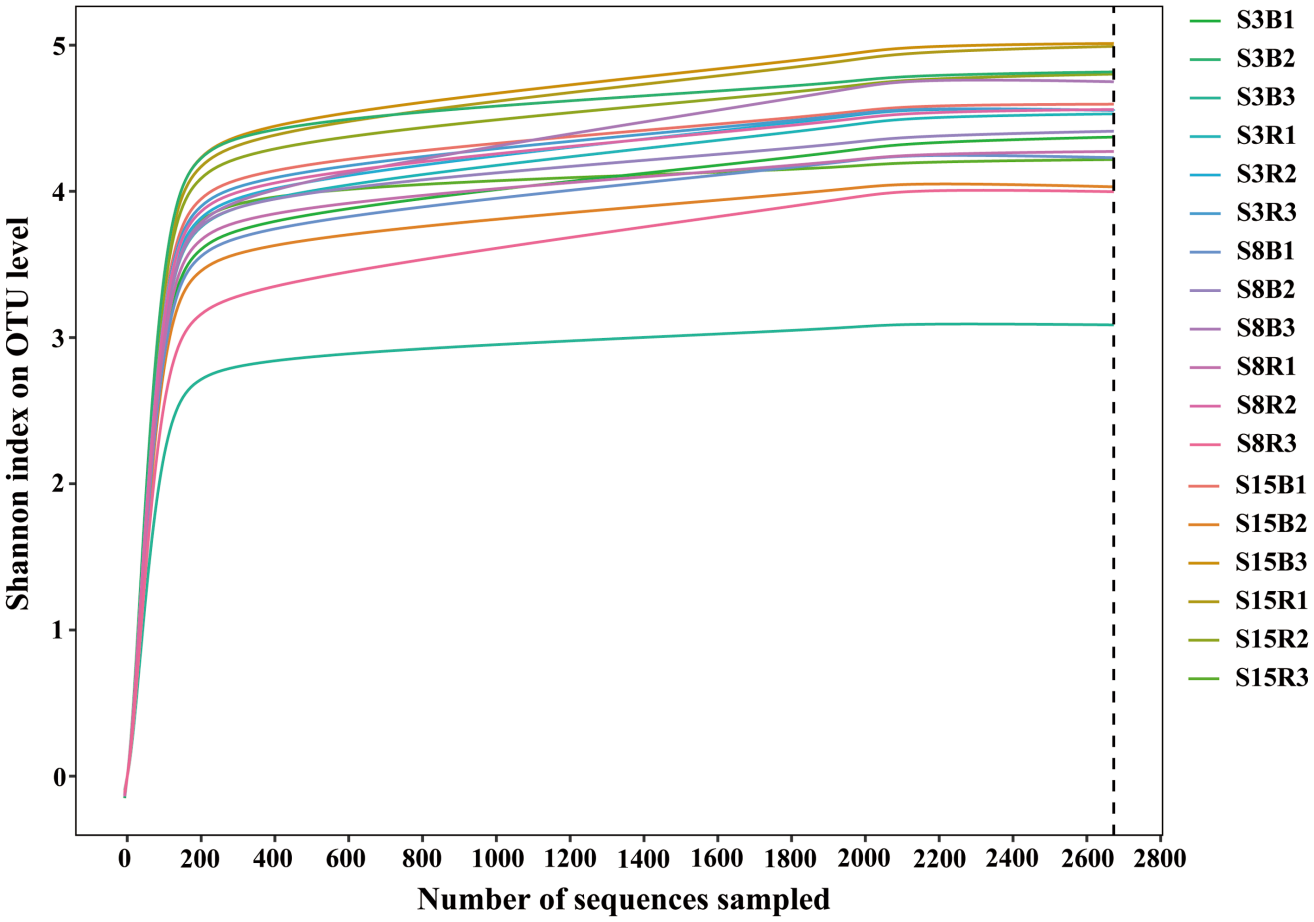
Rarefaction curves of all soil samples. Rarefaction curves of OTUs were clustered for a dissimilarity threshold of 3%. S3B, S3R, S8B, S8R, S15B, and S15R represent non-rhizosphere and rhizosphere soil samples from 3-year-old, 8-year-old, and 15-year-old peach trees, respectively. Each sample had three replicates.

Sample abbreviations are as in Figure 1. Climate factors: Average annual precipitation (PRECTP), Average annual temperature (TEMP), Average annual land surface temperature (LST), Average annual relative humidity (RH), The annual average evaporation land (EVLAND). The values of mean ± SE (standard error) of meteorological data of Shihezi region in the past 10 years are shown in the table.

Non-rhizosphere and rhizosphere soils were collected from peach trees of 3, 8 and 15 years of age, respectively. Non-rhizosphere soil samples, i.e., bulk soil samples (S3B, S8B, S15B) were collected at a distance of 1 m from the main trunk and at a depth of 30 cm, while rhizosphere soil samples (S3R, S8R, S15R) were collected at a distance of 30 cm from the main trunk and at a depth of 30 cm. Samples were collected using a five-point sampling method. Specifically, five peach trees each of three, eight, and fifteen years of age were randomly selected, and non-rhizosphere and rhizosphere soils were collected from each tree separately according to the previously described requirements, and plant residues and stones were removed with a shovel and sieve. Each of the five soil samples of non-rhizosphere and rhizosphere soils from the same tree age was then homogeneously mixed and divided into three equal parts, respectively. Both non-rhizosphere and rhizosphere soil samples have three replicates for each tree age, for a total of 18 samples. Samples were collected at the fruiting stage. Each sample was stored individually in sterile self-sealing bags and transported to the laboratory immediately, then filtered with a 2 mm sieve. They were divided into two parts: one part stored at room temperature for soil physicochemical analysis; the other part was stored in a −20°C refrigerator for subsequent DNA extraction.

### 2.2. DNA extraction and PCR amplification

PowerSoil® DNA Isolation Kit (MoBio Laboratories) was used to extract total DNA in triplicate from soil samples (0.25 g) by using manufacturer’s protocol. The DNA extractions from the same soil sample were combined, then quantified using a NanoDrop 2000 UV–Vis spectrophotometer (Thermo Scientific, United States). The integrity of the DNA was detected using 0.8% agarose gel electrophoresis. The purity and amount of DNA is shown in Supplementary Table S1. The region D1 of the LSU rRNA gene was amplified with a pair of specific primers with barcode NL1F (5′-GCATATCAATAAGCGGAGGAAAAG-3′) and NL2R (5′-CTTGTTCGCTATCGGTCTC-3′; Liu et al., 2018). The PCR reaction system were performed in 20 μL volume containing 5× Fast*Pfu* Buffer (4 μL), 5 μM forward primer and reverse primer (0.8 μL each), 2.5 mM dNTPs (2 μL), 0.4 μL Fast*Pfu* Polymerase, 0.2 μL BSA, and 10 ng of the template DNA. An equal amount of sterile water instead of template DNA was used as a negative control. Amplification was initiated with 5 min at 98°C, followed by 30 cycles of denaturation at 98°C for 30 s, primer annealing at 52°C for 30 s, extension at 72°C for 45 min, and final extension for 5 min at 72°C. Reactions, performed in triplicate, were combined. The PCR products were purified by using 2% agarose gel electrophoresis followed by the AxyPrep DNA Gel Extraction Kit (Axygen Biosciences, United States) and quantified using QuantiFluor™-ST (Promega, United States; Zhang et al., 2018). High-throughput sequencing with the Illumina MiSeq PE300 platform (Illumina, United States) was performed by using paired-end sequencing, which follows the instructions by Personal Biotechnology Co., Ltd. (Shanghai, China).

### 2.3. Sequence processing

The raw Illumina sequences were assigned to individual samples based on their unique barcodes. Raw sequence files were then demultiplexed, quality filtered and analyzed by merging Paired-end reads with FLASH and the Quantitative Insight Into Microbial Ecology (QIIME) software package, respectively (Caporaso et al., 2010; Magoč and Salzberg, 2011). Operational taxonomic unit (OTU) were clustered using UPARSE (version 7.1; http://drive5.com/uparse/) with a threshold of 97% pairwise identity (Edgar, 2013). UCHIME software was used to identify and remove the chimeric sequences. OTUs were classified taxonomically using the Ribosomal Database Project (RDP) classifier (version 2.2; http://sourceforge.net/pro-jects/rdp-classifier/; Wang et al., 2007) against the database of National Centre for Biotechnology Information (NCBI; National Centre for Biotechnology Information, https://www.ncbi.nlm.nih.gov/public/). Any OTUs representing non-yeast sequences were removed before down-stream analysis (Bokulich et al., 2013).

### 2.4. Determination of soil physical and chemical properties

The soil water suspension was shaken for 30 min and then measured by a glass electrode meter for pH value. Conductivity (CO) was measured by electrode method after mixing a naturally dried soil sample with water at a ratio of 1:5 (M/V). Soil water content (SWC) was measured by the drying method, where a moist soil sample of known weight is dried in an oven and then weighed, and the moisture lost by heating represents the soil moisture in the moist sample. Organic matter (OM) was measured by titration with ferrous sulfate, using o-phenanthroline as the indicator. The total nitrogen (TN) was determined by the Kjeldahl method. Total phosphorus (TP) and total potassium (TK) were measured by acid solubilization (Bao, 2007).

### 2.5. Data analysis

The observed richness (Sobs), ACE index, Chao1 estimator, Shannon diversity index and Simpson diversity index of the samples were calculated using QIIME (Caporaso et al., 2010). And the rarefaction curve was plotted based on the diversity index using the “vegan” and “ggplot2” packages in R (v4.3.0). Alpha diversity and soil physical and chemical properties were compared between samples by SPSS Statistics v25.0 software (IBM, United States) based on Kruskal-Wallis test. All values are presented as mean ± standard error (mean ± SE). Differences were taken statistically significant at *p* < 0.05. The Venn diagram was drawn using the “VennDiagram” package and the community bar graph was plotted using “ggplot2” and “ggalluvial” packages in R (v4.2.1). Heatmap were created based on the “vegan” and “pheatmap” packages in R (v4.3.0) to analyze the differences in dominant genera across samples. Principal co-ordinate analysis (PCoA) was done based on Bray-Curtis at OTU level to analyze similarities or differences in the community composition of samples using “vegan” and “ape” packages in R (v4.3.0). Tests for differences between groups in PCoA were analyzed using ANOSIM (analysis of similarities) by vegan package in R. Redundancy analysis (RDA) was used to evaluate the relationships between soil factors and yeast communities, and the plot was drawn by the “vegan” and “ggplot2” packages in R (v4.3.0). Construct a co-occurrence network for each sample group based on the absolute abundance of OTUs. Network topological properties were calculated using the “igraph” package in R (v4.3.0). To reduce network complexity and ensure network reliability, the co-occurrence networks at the genus level were constructed by retaining OTUs with relative abundance ≥ 0.01% and Spearman’s correlation coefficient |r| ≥ 0.6, with significance *p* < 0.01. The co-occurrence network was visualized using Gephi (v.0.10.0; Barberán et al., 2012; Meng et al., 2022). The node data and edge data files for each sample used to generate the co-occurrence network graphs are shown in Supplementary Tables S2–S13.

## 3. Results

### 3.1. Alpha diversity of yeast

According to the age of the peach trees and the sampling locations, we divided the 18 soil samples into 6 groups, named S3B (3-year-old non-rhizosphere), S3R (3-year-old rhizosphere), S8B (8-year-old non-rhizosphere), S8R (8-year-old rhizosphere), S15B (15-year-old non-rhizosphere), and S15R (15-year-old rhizosphere). Based on high-throughput sequencing of the D1 domain of the large subunit (LSU) rRNA gene, we obtained a total of 968,960 sequences from 18 soil samples after removing chimeras and sequences with low-quality reads, and 48,114 yeast sequence reads after excluding non-yeast sequence reads. All yeast sequence reads were clustered into 3,103 operational taxonomic units (OTUs) based on 97% similarity. The yeast rarefaction curves of all samples tended to be flat, indicating that the sequencing depth of the samples was sufficient and the sampling was reasonable (Figure 1).

**Figure 2:**
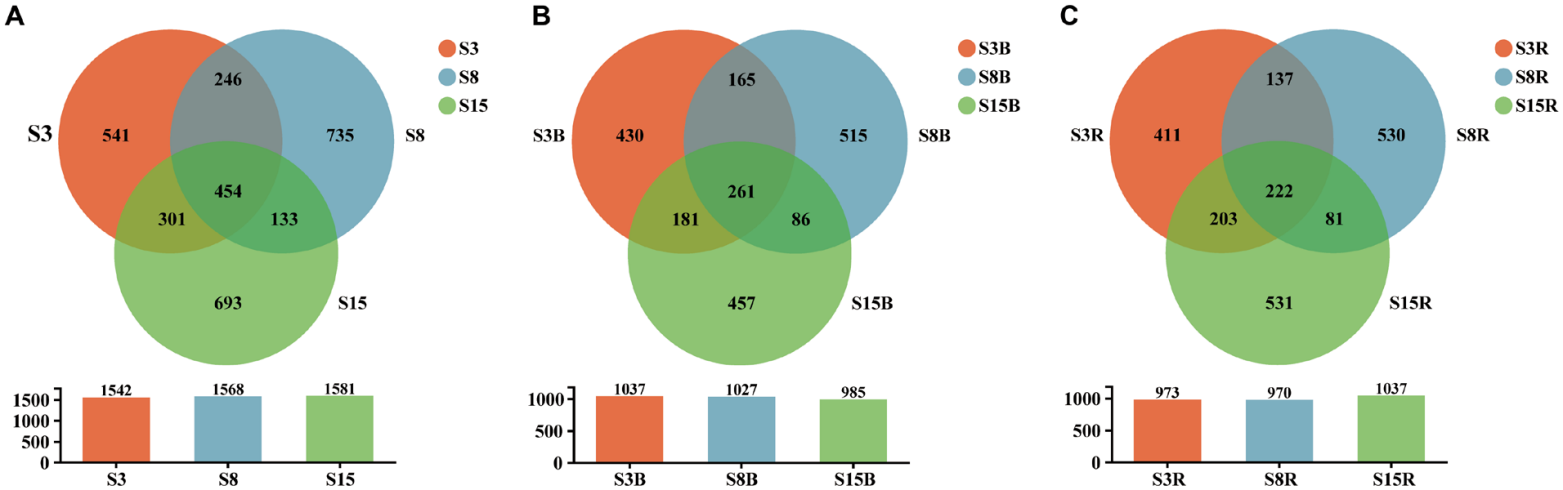
Venn diagram at the OTU level of soil samples from **(A)** non-rhizosphere and rhizosphere of 3-year-old (S3), 8-year-old (S8), and 15-year-old (S15) peach trees, **(B)** non-rhizosphere of 3-year-old (S3B), 8-year-old (S8B), and 15-year-old (S15B) peach trees and **(C)** rhizosphere of 3-year-old (S3R), 8-year-old (S8R), and 15-year-old (S15R) peach trees, respectively. Each circle with different colors in the diagram represents a group; middle core numbers represent the number of OTUs common to all groups. The shared and unique yeast OTUs were shown at a 0.03 dissimilarity distance after removing singletons.

The results of alpha diversity analysis showed that there were no significant differences in the observed species richness (Sobs), species richness (Chao1 and ACE indices) and species diversity (Shannon and Simpson indices; *p* > 0.05; Table 2). The OTUs of S3 (3-year rhizosphere and non-rhizosphere), S8 (8-year rhizosphere and non-rhizosphere) and S15 (15-year rhizosphere and non-rhizosphere) were 1,542, 1,568, and 1,581, respectively. We found that 454 OTUs shared between S3, S8 and S15; 700 OTUs are shared between S3 and S8, 755 OTUs between S3 and S15 and 587 OTUs between S8 and S15. The OTUs of non-rhizosphere (S3B, S8B and S15B) and rhizosphere (S3R, S8R, and S15R) were 2,095 and 2,115, respectively. Among them, the OTUs of S3B, S8B, and S15B were 1,037, 1,027, and 985, respectively; 261 OTUs shared between S3B, S8B, and S15B; 426 OTUs are shared between S3B and S8B, 442 OTUs between S3B and S15B and 347 OTUs between S8B and S15B. The OTUs of S3R, S8R, and S15R were 973, 970, and 1,037, respectively; 222 OTUs shared between S3R, S8R, and S15R; 359 OTUs are shared between S3R and S8R, 425 OTUs between S3R and S15R and 303 OTUs between S8R and S15R (Figure 2).

**Table 2:** Alpha diversity indices of soil samples in peach orchard.

Sample name	Sobs	Chao1	ACE	Shannon	Simpson
S3B	473.0 ± 60.58a	853.5 ± 66.31a	959.1 ± 99.11a	5.903 ± 0.748a	0.884 ± 0.068a
S3R	470.7 ± 10.68a	843.5 ± 59.85a	938.5 ± 89.50a	6.558 ± 0.012a	0.962 ± 0.005a
S8B	486.0 ± 26.98a	806.0 ± 44.66a	899.5 ± 39.75a	6.440 ± 0.220a	0.953 ± 0.008a
S8R	460.3 ± 26.98a	973.9 ± 67.71a	1054.1 ± 55.16a	6.170 ± 0.234a	0.935 ± 0.012a
S15B	463.0 ± 54.15a	701.5 ± 135.85a	756.3 ± 154.26a	6.559 ± 0.410a	0.949 ± 0.021a
S15R	483.3 ± 36.17a	852.9 ± 81.02a	891.4 ± 84.55a	6.738 ± 0.336a	0.963 ± 0.011a

Samples abbreviations are as in Figure 1. Each sample had three replicates. Sobs index was the observed species richness, Chao1 and ACE indices were used to evaluate species richness, Shannon and Simpson indices were used to evaluate species diversity. The values of mean ± SE (standard error) of three samples are shown in the table. Different lowercase letters indicate a significant difference between groups, while the same lowercase letter indicates no significant difference between groups (Kruskal-Wallis test, *p* < 0.05).

### 3.2. Yeast community structure in the soil

Next, 3,103 OTUs were identified as two phyla (Ascomycota and Basidiomycota) and 114 genera. Ascomycota contained 60 genera accounting for 44.78% of all yeast sequences, and Basidiomycota had 54 genera accounting for 55.22% (Tables 3, 4). These include 19 dominant genera that accounted for greater than 1% were *Cryptococcus* (21.40%), *Pichia* (9.615%), *Clavispora* (9.249%), *Tausonia* (5.491%), *Zygosaccharomyces* (4.849%), *Solicoccozyma* (4.527%), *Udeniomyces* (3.912%), *Candida* (3.294%), *Filobasidium* (3.284%), *Trigonopsis* (3.124%), *Aureobasidium* (3.024%), *Papiliotrema* (2.822%), *Saturnispora* (2.752%), *Rhodotorula* (2.060%)*, Saitozyma* (2.058%), *Cyniclomyces* (1.567%), *Goffeauzyma* (1.814%), *Naganishia* (1.594%), and *Cyberlindnera* (1.122%). The 19 dominant genera accounted for 87.558% of all yeast sequences. In addition, a total of 15 rare yeast genera (genus with less than 10% frequency of occurrence) were included in all soil samples, accounting for approximately 13.16% of all yeast genera. 33 yeast genera were shared by 18 soil samples, and 24 yeast genera were significantly different among S3B, S3R, S8B, S8R, S15B, and S15R groups (*p* < 0.05; Tables 3, 4).

**Table 3:** The percentage and frequency of occurrence of Ascomycetous yeasts (accounted for 44.78%) in all samples.

**No.**	Genus	Sample name						Total^a^ (%)	Occurrence frequency (%)
		S3B (%)	S3R (%)	S8B (%)	S8R (%)	S15B (%)	S15R (%)		
1	*Pichia*	20.23 ± 0.167a	6.310 ± 0.018a	4.539 ± 0.026a	14.85 ± 0.116a	6.559 ± 0.029a	5.200 ± 0.038a	9.615	100
2	*Clavispora*	19.17 ± 0.103a	11.77 ± 0.017a	5.263 ± 0.011a	5.300 ± 0.004a	8.991 ± 0.020a	5.000 ± 0.006a	9.249	100
3^c^	*Zygosaccharomyces*	2.569 ± 0.024b	0.062 ± 0.001b	13.97 ± 0.015a	12.00 ± 0.001a	0.212 ± 0.001b	0.287 ± 0.001b	4.849	100
4	*Candida*	2.419 ± 0.004a	1.546 ± 0.004a	2.008 ± 0.002a	2.993 ± 0.004a	7.333 ± 0.047a	3.467 ± 0.011a	3.294	100
5	*Trigonopsis*	2.818 ± 0.007a	1.746 ± 0.001a	2.581 ± 0.013a	1.460 ± 0.007a	3.080 ± 0.005a	7.058 ± 0.049a	3.124	100
6^c^	*Aureobasidium*	2.058 ± 0.005b	2.020 ± 0.004b	2.270 ± 0.003b	2.245 ± 0.004b	5.487 ± 0.002a	4.065 ± 0.018ab	3.024	100
7	*Saturnispora*	0.399 ± 0.002a	1.434 ± 0.009a	1.609 ± 0.009a	0.873 ± 0.004a	10.38 ± 0.016a	1.821 ± 0.016a	2.752	100
8^c^	*Cyniclomyces*	0.037 ± 0.001b	0.200 ± 0.002b	8.667 ± 0.043a	0.486 ± 0.002b	0.012 ± 0.001b	-	1.567	50
9	*Cyberlindnera*	0.075 ± 0.001a	0.224 ± 0.001a	-	0.025 ± 0.001a	0.137 ± 0.001a	6.273 ± 0.062a	1.122	66.7
10	*Hyphopichia*	0.860 ± 0.007a	0.761 ± 0.005a	0.611 ± 0.003a	1.322 ± 0.002a	0.200 ± 0.001a	0.661 ± 0.005a	0.736	100
11	*Meyerozyma*	1.097 ± 0.005a	0.299 ± 0.001a	0.324 ± 0.001a	0.362 ± 0.001a	0.449 ± 0.001a	1.434 ± 0.009a	0.661	100
12^c^	*Metschnikowia*	0.274 ± 0.001b	0.200 ± 0.001b	0.362 ± 0.001b	0.200 ± 0.001b	0.237 ± 0.013b	2.21 ± 0.003a	0.580	100
13^c^	*Yamadazyma*	0.549 ± 0.003b	0.175 ± 0.001b	0.112 ± 0.001b	0.237 ± 0.001b	1.372 ± 0.002a	0.337 ± 0.001b	0.463	100
14	*Blastobotrys*	0.524 ± 0.002a	0.536 ± 0.002a	0.108 ± 0.001a	0.150 ± 0.001a	0.287 ± 0.001a	0.811 ± 0.004a	0.401	94.4
15^c^	*Geotrichum*	0.125 ± 0.001b	0.274 ± 0.001b	0.010 ± 0.001b	0.150 ± 0.001b	0.412 ± 0.002b	0.935 ± 0.002a	0.333	94.4
16	*Collophora*	0.187 ± 0.001a	0.274 ± 0.002a	0.187 ± 0.001a	0.461 ± 0.002a	0.374 ± 0.001a	0.461 ± 0.001a	0.324	100
17^c^	*Wickerhamomyces*	0.187 ± 0.001b	0.112 ± 0.001b	0.050 ± 0.001b	0.212 ± 0.002b	0.187 ± 0.001b	1.110 ± 0.005a	0.310	94.4
18	*Eremothecium*	0.412 ± 0.001a	0.349 ± 0.001a	0.299 ± 0.002a	0.287 ± 0.001a	0.287 ± 0.001a	0.187 ± 0.001a	0.303	100
19	*Galactomyces*	0.137 ± 0.001a	0.125 ± 0.001a	-	0.112 ± 0.001a	0.112 ± 0.001a	1.097 ± 0.010a	0.264	55.6
20	*Kazachstania*	0.200 ± 0.001a	0.101 ± 0.001a	0.212 ± 0.001a	0.012 ± 0.001a	0.599 ± 0.006a	0.150 ± 0.001a	0.212	83.3
21	*Tetrapisispora*	0.025 ± 0.001a	0.012 ± 0.001a	0.175 ± 0.001a	0.960 ± 0.008a	0.050 ± 0.001a	0.025 ± 0.001a	0.208	61.1
22^c^	*Lachancea*	0.037 ± 0.001b	0.137 ± 0.001ab	0.050 ± 0.001b	0.200 ± 0.001ab	0.474 ± 0.002a	0.050 ± 0.001b	0.158	77.8
23	*Schizosaccharomyces*	0.162 ± 0.001a	0.100 ± 0.001a	0.212 ± 0.001a	0.037 ± 0.001a	0.010 ± 0.002a	0.249 ± 0.002a	0.143	88.9
24	*Nakaseomyces*	0.324 ± 0.003a	0.012 ± 0.001a	0.112 ± 0.000a	0.112 ± 0.001a	-	0.050 ± 0.002a	0.102	55.6
25^**c**^	*Issatchenkia*	0.087 ± 0.001b	0.037 ± 0.001b	0.012 ± 0.000b	0.025 ± 0.001b	0.387 ± 0.001a	0.025 ± 0.001b	0.096	66.7
26	*Starmerella*	0.012 ± 0.001a	0.237 ± 0.002a	0.012 ± 0.001a	0.025 ± 0.001a	0.050 ± 0.001a	0.150 ± 0.001a	0.081	61.1
27^c^	*Danielozyma*	-	0.050 ± 0.001b	0.037 ± 0.001b	0.125 ± 0.001ab	0.212 ± 0.001a	0.037 ± 0.001b	0.077	66.7
28^**c**^	*Millerozyma*	0.162 ± 0.001a	0.087 ± 0.001ab	0.037 ± 0.001b	-	0.012 ± 0.001b	0.150 ± 0.001a	0.075	66.7
29	*Hanseniaspora*	0.087 ± 0.001a	0.087 ± 0.001a	0.101 ± 0.001a	0.037 ± 0.001a	0.075 ± 0.001a	0.012 ± 0.001a	0.067	55.6
30	*Wickerhamiella*	0.062 ± 0.001a	0.012 ± 0.001a	0.050 ± 0.001a	0.012 ± 0.001a	0.012 ± 0.001a	0.150 ± 0.001a	0.050	55.6
31	*Ogataea*	0.025 ± 0.001a	0.162 ± 0.001a	0.037 ± 0.001a	0.012 ± 0.001a	0.037 ± 0.001a	0.012 ± 0.001a	0.048	50
32	*Magnusiomyces*	0.150 ± 0.001a	-	0.062 ± 0.001a	-	-	0.050 ± 0.001a	0.044	22.2
33	*Zygoascus*	0.037 ± 0.001ab	0.012 ± 0.001ab	-	-	0.012 ± 0.001ab	0.187 ± 0.001a	0.042	38.9
34^c^	*Saccharomyces*	-	0.050 ± 0.001b	-	0.150 ± 0.001a	0.012 ± 0.001b	0.037 ± 0.001b	0.042	44.4
35	*Kluyveromyces*	0.062 ± 0.001a	-	0.037 ± 0.001a	0.025 ± 0.001a	0.075 ± 0.001a	0.050 ± 0.001a	0.042	50
36	*Sugiyamaella*	0.012 ± 0.001a	0.012 ± 0.001a	0.025 ± 0.001a	0.075 ± 0.001a	-	0.075 ± 0.001a	0.033	38.9
37	*Middelhovenomyces*	0.037 ± 0.001a	0.025 ± 0.001a	0.062 ± 0.001a	0.012 ± 0.001a	0.012 ± 0.001a	0.025 ± 0.001a	0.029	50
38	*Schwanniomyces*	0.012 ± 0.001a	0.012 ± 0.001a	0.062 ± 0.001a	0.037 ± 0.001a	0.037 ± 0.001a	-	0.027	38.9
39	*Torulaspora*	0.025 ± 0.001ab	-	-	0.012 ± 0.001ab	-	0.101 ± 0.001a	0.023	22.2
40	*Vanderwaltozyma*	0.037 ± 0.001a	-	0.050 ± 0.001a	-	0.050 ± 0.001a	-	0.023	27.8
41	*Debaryomyces*	-	0.050 ± 0.001a	0.050 ± 0.001a	-	0.012 ± 0.001a	0.012 ± 0.001a	0.021	33.3
42	*Trichomonascus*	0.062 ± 0.001a	0.025 ± 0.001a	0.012 ± 0.001a	0.012 ± 0.001a	0.012 ± 0.001a	-	0.021	33.3
43	*Spencermartinsiella*	0.050 ± 0.001a	0.037 ± 0.001a	-	0.012 ± 0.001a	-	0.012 ± 0.001a	0.019	27.8
44	*Zygotorulaspora*	0.012 ± 0.001a	0.012 ± 0.001a	0.037 ± 0.001a	0.025 ± 0.001a	-	0.012 ± 0.001a	0.017	38.9
45	*Kurtzmaniella*	-	0.025 ± 0.001a	0.025 ± 0.001a	0.025 ± 0.001a	-	0.012 ± 0.001a	0.015	27.8
46	*Saccharomycodes*	-	0.012 ± 0.001a	-	0.025 ± 0.001a	0.037 ± 0.001a	0.012 ± 0.001a	0.015	33.3
47	*Naumovozyma*	-	0.012 ± 0.001ab	-	-	0.012 ± 0.001ab	0.062 ± 0.001a	0.015	22.2
48	*Kodamaea*	0.012 ± 0.001a	-	-	-	0.037 ± 0.001a	0.012 ± 0.001a	0.010	16.7
49	*Scheffersomyces*	0.012 ± 0.001a	0.037 ± 0.001a	-	-	0.012 ± 0.001a	-	0.010	16.7
50	*Tortispora*	-	-	0.012 ± 0.001a	-	0.012 ± 0.001a	0.025 ± 0.001a	0.008	22.2
51	*Barnettozyma*	-	-	-	0.012 ± 0.001a	0.012 ± 0.001a	0.012 ± 0.001a	0.006	16.7
52^b^	*Spathaspora*	-	-	0.025 ± 0.001a	-	-	-	0.004	5.56
53^b^	*Lipomyces*	0.025 ± 0.001a	-	-	-	-	-	0.004	5.56
54^b^	*Sporopachydermia*	-	0.012 ± 0.001a	-	-	0.012 ± 0.001a	-	0.004	5.56
55^b^	*Citeromyces*	0.012 ± 0.001a	0.012 ± 0.001a	-	-	-	-	0.004	11.1
56^b^	*Macrorhabdus*	-	-	-	-	-	0.025 ± 0.001a	0.004	5.56
57^b^	*Nielozyma*	-	-	-	0.025 ± 0.001a	-	-	0.004	5.56
58^b^	*Myxozyma*	0.012 ± 0.001a	-	-	-	-	-	0.002	5.56
59^b^	*Nakazawaea*	-	-	-	-	0.012 ± 0.001a	-	0.002	5.56
60^b^	*Nadsonia*	-	-	-	0.012 ± 0.001a	-	-	0.002	5.56

“-” indicates a value of 0. ^a^Percentage of sequence reads for the yeast genus in all samples, ^b^rare yeasts of the genera (genus with an occurrence frequency of less than 10% in all samples), ^c^Yeast genera with significantly different proportions in the different samples. Different lowercase letters indicate a significant difference between groups, while the same lowercase letter indicates no significant difference between groups (Kruskal-Wallis test, *P* < 0.05).

**Table 4:** The percentage and frequency of occurrence of Basidiomycetes yeasts (accounted for 55.22%) in all samples.

**No.**	Genus	Sample name						Total^a^ (%)	Occurrence Frequency (%)
		S3B (%)	S3R (%)	S8B (%)	S8R (%)	S15B (%)	S15R (%)		
1	*Cryptococcus*	15.29 ± 0.026a	28.25 ± 0.022a	28.68 ± 0.085a	27.12 ± 0.080a	11.62 ± 0.005a	17.45 ± 0.027a	21.40	100
2^**c**^	*Tausonia*	3.467 ± 0.010b	8.206 ± 0.149a	3.629 ± 0.001b	3.542 ± 0.015b	7.158 ± 0.009a	6.946 ± 0.002a	5.491	100
3	*Solicoccozyma*	6.934 ± 0.049a	2.968 ± 0.004a	2.095 ± 0.003a	3.217 ± 0.015a	5.711 ± 0.016a	6.235 ± 0.003a	4.527	100
4^c^	*Udeniomyces*	2.943 ± 0.008b	4.651 ± 0.005ab	3.367 ± 0.005ab	3.192 ± 0.006ab	4.377 ± 0.005ab	4.938 ± 0.004a	3.912	100
5	*Filobasidium*	2.407 ± 0.005a	1.596 ± 0.003a	5.026 ± 0.003a	4.427 ± 0.005a	5.163 ± 0.040a	1.085 ± 0.001a	3.284	100
6^**c**^	*Papiliotrema*	1.733 ± 0.004ab	3.093 ± 0.003ab	4.015 ± 0.012a	3.143 ± 0.006ab	3.729 ± 0.011ab	1.222 ± 0.005b	2.822	100
7	*Rhodotorula*	0.536 ± 0.001a	7.956 ± 0.063a	0.387 ± 0.001a	0.249 ± 0.001a	2.170 ± 0.010a	1.060 ± 0.003a	2.060	100
8	*Saitozyma*	1.334 ± 0.004a	3.292 ± 0.012a	1.322 ± 0.001a	1.895 ± 0.003a	2.594 ± 0.008a	1.908 ± 0.006a	2.058	100
9	*Goffeauzyma*	1.958 ± 0.007a	1.858 ± 0.003a	1.671 ± 0.003a	1.197 ± 0.004a	1.496 ± 0.004a	2.706 ± 0.008a	1.814	100
10^c^	*Naganishia*	1.122 ± 0.003b	1.858 ± 0.002ab	1.072 ± 0.001b	0.923 ± 0.001b	1.322 ± 0.001b	3.267 ± 0.013a	1.594	100
11	*Mrakia*	0.910 ± 0.003a	1.085 ± 0.001a	0.436 ± 0.001a	0.848 ± 0.002a	0.748 ± 0.001a	1.584 ± 0.010a	0.935	100
12^c^	*Cystofilobasidium*	0.823 ± 0.003ab	1.110 ± 0.003ab	0.486 ± 0.002b	0.549 ± 0.002ab	0.611 ± 0.002ab	1.496 ± 0.005a	0.846	100
13^**c**^	*Cystobasidium*	0.910 ± 0.005ab	0.723 ± 0.002ab	0.137 ± 0.001c	0.661 ± 0.002ab	1.122 ± 0.002a	0.224 ± 0.001bc	0.630	100
14	*Trichosporon*	0.349 ± 0.001a	0.436 ± 0.001a	0.120 ± 0.001a	0.262 ± 0.001a	0.486 ± 0.002a	1.684 ± 0.012a	0.569	100
15^c^	*Apiotrichum*	0.673 ± 0.001a	0.137 ± 0.001b	0.436 ± 0.002ab	0.474 ± 0.001ab	0.175 ± 0.001bc	0.120 ± 0.001bc	0.349	100
16	*Derxomyces*	0.387 ± 0.001a	0.324 ± 0.001a	0.474 ± 0.001a	0.412 ± 0.001a	0.212 ± 0.001a	0.262 ± 0.001a	0.345	100
17	*Vishniacozyma*	0.362 ± 0.002a	0.212 ± 0.002a	0.362 ± 0.001a	0.349 ± 0.002a	0.399 ± 0.002a	0.162 ± 0.001a	0.308	94.4
18^c^	*Kwoniella*	0.162 ± 0.001b	0.412 ± 0.002ab	0.262 ± 0.001ab	0.150 ± 0.001b	0.524 ± 0.001a	0.324 ± 0.002ab	0.306	100
19	*Piskurozyma*	0.249 ± 0.001a	0.262 ± 0.001a	0.162 ± 0.001a	0.137 ± 0.001a	0.125 ± 0.001a	0.524 ± 0.003a	0.243	88.9
20	*Xanthophyllomyces*	0.224 ± 0.001a	0.262 ± 0.001a	0.162 ± 0.001a	0.137 ± 0.001a	0.262 ± 0.001a	0.125 ± 0.001a	0.195	100
21	*Carlosrosaea*	0.120 ± 0.001a	0.162 ± 0.001a	0.112 ± 0.001a	0.175 ± 0.001a	0.212 ± 0.001a	0.237 ± 0.001a	0.183	100
22	*Heterocephalacria*	0.162 ± 0.001a	0.162 ± 0.001a	0.112 ± 0.001a	0.112 ± 0.001a	0.120 ± 0.001a	0.324 ± 0.002a	0.179	100
23	*Cutaneotrichosporon*	0.012 ± 0.001a	0.137 ± 0.001a	0.037 ± 0.000a	0.012 ± 0.001a	0.374 ± 0.003a	0.412 ± 0.003a	0.164	61.1
24	*Sporobolomyces*	0.137 ± 0.001a	0.436 ± 0.003a	0.037 ± 0.001a	0.062 ± 0.001a	0.125 ± 0.001a	0.075 ± 0.001a	0.145	77.8
25	*Malassezia*	0.324 ± 0.002a	0.010 ± 0.001a	0.010 ± 0.001a	0.062 ± 0.001a	0.062 ± 0.001a	0.212 ± 0.001a	0.143	83.3
26	*Kurtzmanomyces*	0.037 ± 0.001a	0.087 ± 0.001a	0.112 ± 0.001a	0.287 ± 0.002a	0.062 ± 0.001a	0.062 ± 0.001a	0.108	72.2
27^c^	*Sterigmatomyces*	0.050 ± 0.001b	-	0.187 ± 0.001a	0.224 ± 0.001a	0.012 ± 0.001b	0.125 ± 0.001ab	0.100	61.1
28	*Bullera*	0.062 ± 0.001a	0.062 ± 0.001a	0.062 ± 0.001a	0.050 ± 0.001a	0.062 ± 0.001a	0.137 ± 0.001a	0.073	94.4
29	*Sympodiomycopsis*	0.012 ± 0.001a	0.087 ± 0.001a	0.012 ± 0.001a	0.012 ± 0.001a	0.050 ± 0.001a	0.010 ± 0.001a	0.046	44.4
30	*Erythrobasidium*	0.012 ± 0.001a	0.050 ± 0.001a	0.025 ± 0.001a	0.050 ± 0.001a	0.050 ± 0.001a	0.075 ± 0.001a	0.044	38.9
31^**c**^	*Cystobasidiomycetes*	0.012 ± 0.001b	0.025 ± 0.001ab	0.075 ± 0.000ab	0.025 ± 0.001ab	0.012 ± 0.001b	0.087 ± 0.001a	0.039	66.7
32	*Fereydounia*	0.012 ± 0.001ab	0.025 ± 0.001ab	-	0.012 ± 0.001ab	0.050 ± 0.001ab	0.137 ± 0.001a	0.039	50
33^c^	*Bannoa*	0.012 ± 0.001b	-	0.012 ± 0.001b	0.025 ± 0.001b	-	0.162 ± 0.001a	0.035	33.3
34	*Curvibasidium*	-	-	0.075 ± 0.001a	0.075 ± 0.001a	-	-	0.025	27.8
35	*Tsuchiyaea*	-	0.050 ± 0.001a	0.025 ± 0.001a	0.062 ± 0.001a	-	0.012 ± 0.001a	0.025	38.9
36	*Vanrija*	0.087 ± 0.001a	-	0.012 ± 0.001a	0.025 ± 0.001a	-	0.012 ± 0.001a	0.023	27.8
37^c^	*Hannaella*	0.012 ± 0.001b	0.025 ± 0.001ab	-	0.012 ± 0.001b	0.075 ± 0.001a	0.012 ± 0.001b	0.023	38.9
38	*Trichosporonoides*	0.062 ± 0.001a	0.025 ± 0.001ab	0.025 ± 0.001ab	-	-	-	0.019	33.3
39	*Occultifur*	-	-	-	0.012 ± 0.001a	0.025 ± 0.001a	0.062 ± 0.001a	0.017	22.2
40	*Dioszegia*	0.037 ± 0.001a	-	0.012 ± 0.001a	0.012 ± 0.001a	-	0.025 ± 0.001a	0.015	22.2
41	*Sakaguchia*	-	-	0.012 ± 0.001a	-	0.037 ± 0.001a	0.037 ± 0.001a	0.015	22.2
42	*Cystobasidiopsis*	0.012 ± 0.001a	0.037 ± 0.001a	-	-	0.012 ± 0.001a	0.012 ± 0.001a	0.012	27.8
43	*Sterigmatosporidium*	-	-	-	-	-	0.075 ± 0.001a	0.012	5.56
44	*Acaromyces*	0.025 ± 0.001a	-	-	-	0.037 ± 0.001a	-	0.010	16.7
45	*Microbotryozyma*	-	0.037 ± 0.001a	-	-	0.012 ± 0.001a	-	-	11.1
46	*Ballistosporomyces*	0.037 ± 0.001a	-	-	-	-	-	0.006	5.56
47	*Symmetrospora*	0.012 ± 0.001a	-	-	-	0.025 ± 0.001a	-	0.006	11.1
48	*Kondoa*	-	-	-	0.012 ± 0.001a	0.012 ± 0.001a	-	0.004	11.1
49^b^	*Chionosphaera*	-	-	-	0.025 ± 0.001a	-	-	0.004	5.56
50^b^	*Rhodosporidiobolus*	-	-	0.012 ± 0.001a	-	-	-	0.002	5.56
51^b^	*Meira*	0.012 ± 0.001a	-	-	-	-	-	0.002	5.56
52^b^	*Buckleyzyma*	-	-	-	0.012 ± 0.001a	-	-	0.002	5.56
53^b^	*Langdonia*	-	-	-	-	-	0.012 ± 0.001a	0.002	5.56
54^b^	*Leucosporidium*	-	-	-	0.012 ± 0.001a	-	-	0.002	5.56

“-” indicates a value of 0. ^a^Percentage of sequence reads for the yeast genus in all samples, ^b^rare yeasts of the genera (genus with an occurrence frequency of less than 10% in all samples), ^c^Yeast genera with significantly different proportions in the different samples. Different lowercase letters indicate a significant difference between groups, while the same lowercase letter indicates no significant difference between groups (Kruskal-Wallis test, *P* < 0.05).

**Figure 3:**
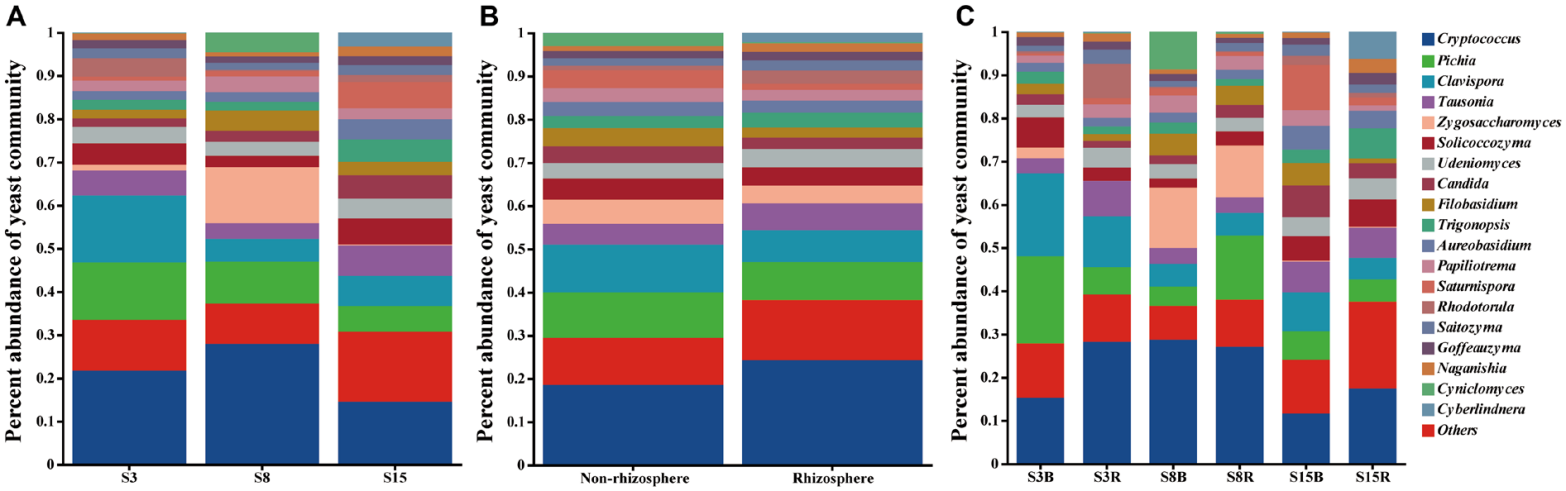
Proportion of dominant yeast genera in **(A)** peach trees of different ages soil samples, **(B)** non-rhizosphere and rhizosphere soil samples, and **(C)** non-rhizosphere and rhizosphere soil samples from peach trees of different ages. Others indicated that yeast genera accounted for less than 1%. Each sample had three replicates (Replicates are not specifically shown in the legend, but have been involved in the analysis). Sample abbreviations are same as presented in Figures 1, 2.

**Figure 4:**
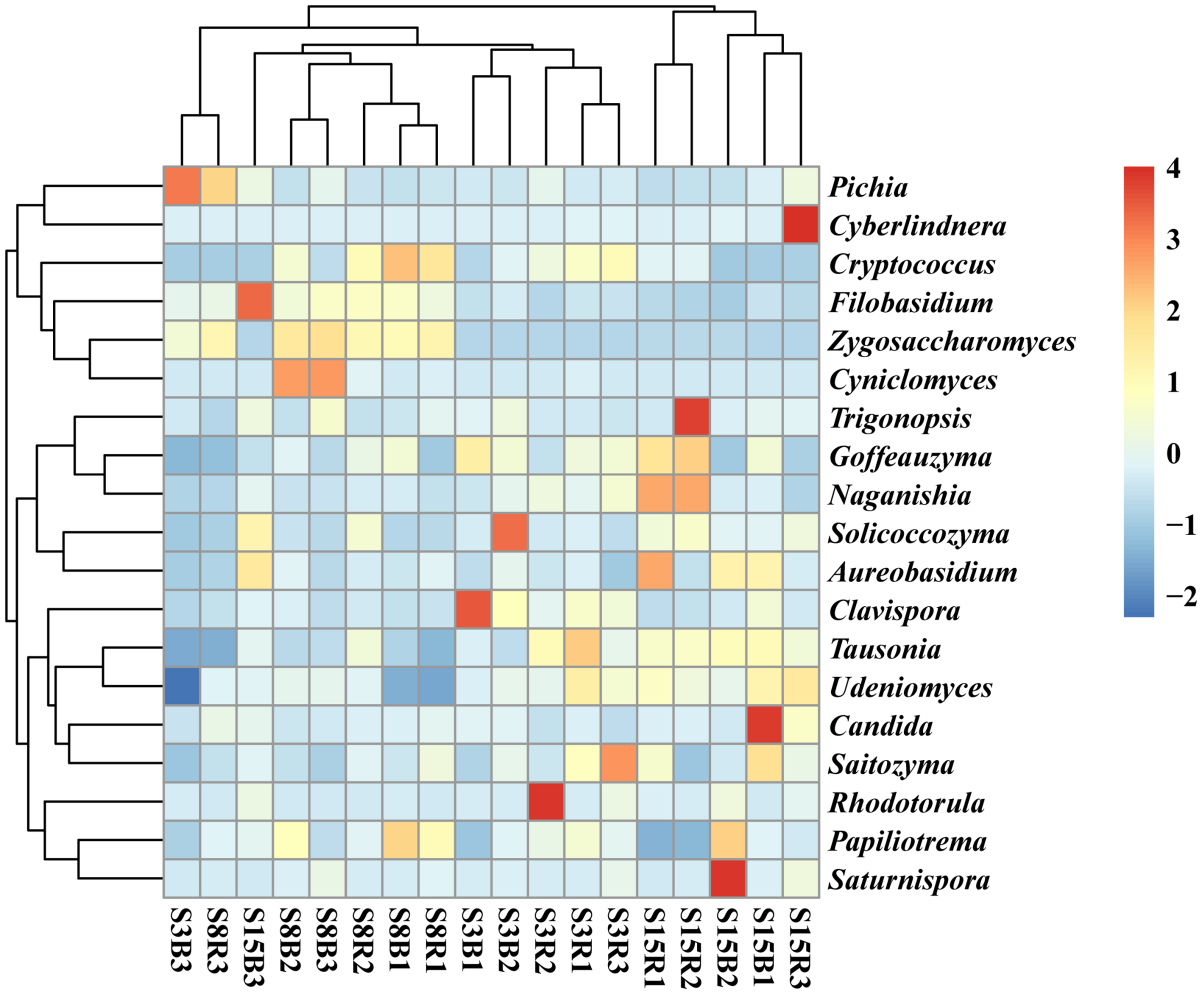
Heatmap of the distribution of the top 19 dominant yeast genera among the different soil samples. The normalized relative abundance of each genus is indicated by a gradient of color from blue (low abundance) to red (high abundance). Sample abbreviations are same as presented in Figure 1.

After that, we show the proportions of 19 dominant genera in each group of soil samples to reveal the differences in yeast community composition between non-rhizosphere and rhizosphere soils of peach trees at different ages (Figure 3). The results showed that the top three dominant genera in S3 were *Cryptococcus* (21.77%), *Clavispora* (15.47%) and *Pichia* (13.27%) in that order; *Cryptococcus* (27.90%), *Zygosaccharomyces* (12.98%) and *Pichia* (9.696%) in S8; and *Cryptococcus* (14.53%), *Tausonia* (7.052%), and *Clavispora* (6.996%) in S15 (Figure 3A). The top three dominant genera in non-rhizosphere soil samples were *Cryptococcus* (18.53%), *Clavispora* (11.14%), and *Pichia* (10.44%) in that order; and *Cryptococcus* (24.27%), *Pichia* (8.787%), and *Clavispora* (7.358%) were found in the rhizosphere soil samples (Figure 3B). Further analysis showed that the top three dominant genera in the soil samples of S3B group were *Pichia* (20.23%), *Clavispora* (19.17%), and *Cryptococcus* (15.29%), in S3R group were *Cryptococcus* (28.25%), *Clavispora* (11.77%), and *Tausonia* (8.206%), in S8B group were *Cryptococcus* (28.68%), *Zygosaccharomyces* (13.97%), and *Cyniclomyces* (8.667%), in S8R group were *Cryptococcus* (27.12%), *Pichia* (14.85%), and *Zygosaccharomyces* (12.00%), S15B soils were *Cryptococcus* (11.62%), *Saturnispora* (10.38%), and *Clavispora* (8.991%), and the top three dominant genera in S15R soils were *Cryptococcus* (17.45%), *Trigonopsis* (7.058%), and *Tausonia* (6.946%), respectively (Figure 3C). And the relative abundances of 7 of the 19 dominant genera differed significantly among the S3B, S3R, S8B, S8R, S15B, and S15R groups, namely *Tausonia*, *Zygosaccharomyces*, *Udeniomyces, Aureobasidium, Papiliotrema, Cyniclomyces,* and *Naganishia* (*p* < 0.05). *Tausonia* and *Udeniomyces* were mainly present in S3R, S15B and S15R, *Zygosaccharomyces* was mainly present in S8B and S8R, *Aureobasidium* was mainly found in S15B and S15R, *Papiliotrema* and *Cyniclomyces* were mainly detected in S8B and *Cyniclomyces* could not be detected in S15R, and *Naganishia* was mainly found in S3R and S15R (Figure 4; Tables 3, 4). In addition, the heat map results largely show that soil samples from the same tree age are clustered together (Figure 4).

### 3.3. Relationship between yeast communities in soil samples of different ages

**Figure 5:**
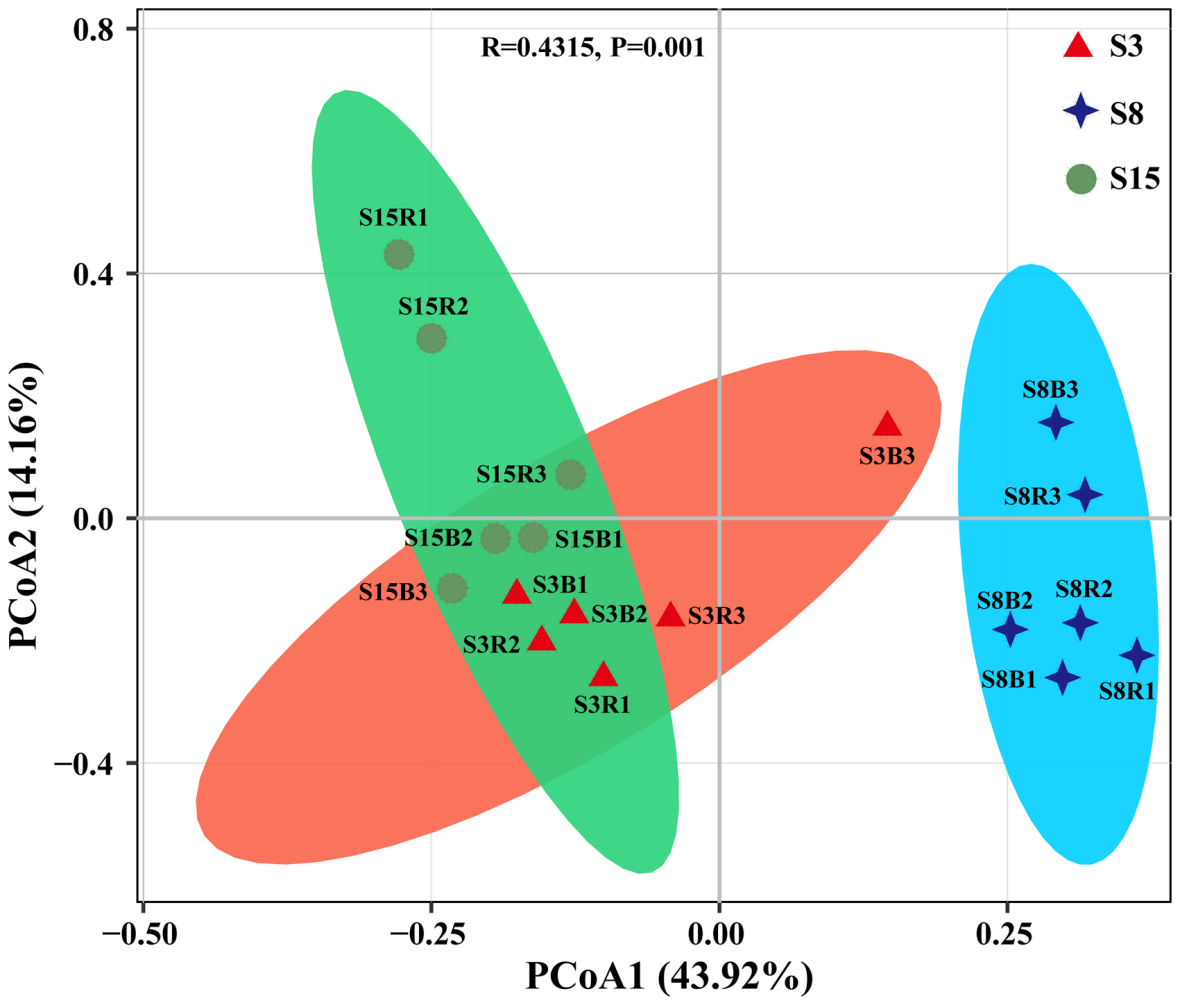
Principal Coordinates analysis (PCoA) based on Bray-Curtis distance method at the OTU level. Red triangles, blue diamonds and green circles represent samples from non-rhizosphere and rhizosphere of 3-year-old (S3), 8-year-old (S8), and 15-year-old (S15) peach trees, respectively. Sample abbreviations are same as presented in Figures 1, 2.

We further evaluated the similarity of the yeast community composition of 18 soil samples based on PCoA (Figure 5). The results showed that the variance explained by the first principal axis (PCoA1) alone was 43.92%, and the variance explained by the second principal axis (PCoA2) alone was 14.16%. In general, the 18 samples were first clustered together according to tree age, followed by clustering according to rhizosphere or non-rhizosphere criteria, only with an overlapping between the samples from the S3 and S15 groups on the score plots, indicating significant differences in community composition between groups, and this result can also be proved by both R-value (0.4315) and *p*-value (*p* = 0.001). S3 and S15 were more similar in community composition for intergroups, and there were significant differences in community composition between rhizosphere or non-rhizosphere samples at the same tree age.

### 3.4. Relationship among soil samples, yeast community structure, and soil factors

In order to clarify the relationship between soil sample similarity, yeast community structure and soil factors, we first examined the soil physical and chemical properties that including soil pH, conductivity (CO), soil water content (SWC), organic matter (OM), total nitrogen (TN), total phosphorus (TP) and total potassium (TK). The analysis of soil physical and chemical properties showed that the pH values were significantly higher in S3B and S8R and significantly lower in S15R than in the rest of the samples; SWC values were significantly higher in S3B and significantly lower in S8R and S15B than in other samples; while the OM, TN and TK contents were significantly higher in S15R than in other samples (*p* < 0.05); the CO and TK levels were not significantly different in all samples (*p* > 0.05; Table 5).

**Table 5:** The soil physical and chemical properties of soil samples in peach orchard.

Sample name	pH	CO (mS/cm)	SWC (%)	OM (g/kg)	TN (g/kg)	TP (g/kg)	TK (g/kg)
S3B	8.02 ± 0.066ab	0.147 ± 0.018a	0.110 ± 0.017a	17.09 ± 2.356b	0.503 ± 0.058b	0.973 ± 0.082b	77.26 ± 19.55a
S3R	8.04 ± 0.029ab	0.163 ± 0.027a	0.071 ± 0.004ab	16.69 ± 2.783b	0.480 ± 0.049b	0.843 ± 0.032b	46.79 ± 18.19a
S8B	8.33 ± 0.204a	0.127 ± 0.009a	0.079 ± 0.016ab	17.32 ± 3.204b	0.507 ± 0.093b	1.750 ± 0.730b	50.03 ± 2.932a
S8R	8.41 ± 0.119a	0.143 ± 0.033a	0.048 ± 0.004b	15.13 ± 0.494b	0.450 ± 0.006b	0.983 ± 0.112b	46.22 ± 13.28a
S15B	7.93 ± 0.052ab	0.140 ± 0.015a	0.040 ± 0.008b	16.35 ± 0.530b	0.497 ± 0.024b	1.013 ± 0.058b	90.48 ± 23.74a
S15R	7.76 ± 0.042b	0.160 ± 0.010a	0.071 ± 0.025ab	31.20 ± 3.533a	1.037 ± 0.097a	3.573 ± 0.543a	53.79 ± 8.837a

Sample abbreviations are as in Figure 1. Each sample had three replicates. Soil physicochemical properties: pH, Conductivity (CO), Soil water content (SWC), Organic matter (OM), Total nitrogen (TN), Total phosphorus (TP), Total potassium (TK). The values of mean ± SE (standard error) of three samples are shown in the table. Different lowercase letters indicate a significant difference between groups, while the same lowercase letter indicates no significant difference between groups (Kruskal-Wallis test, *p* < 0.05).

**Figure 6:**
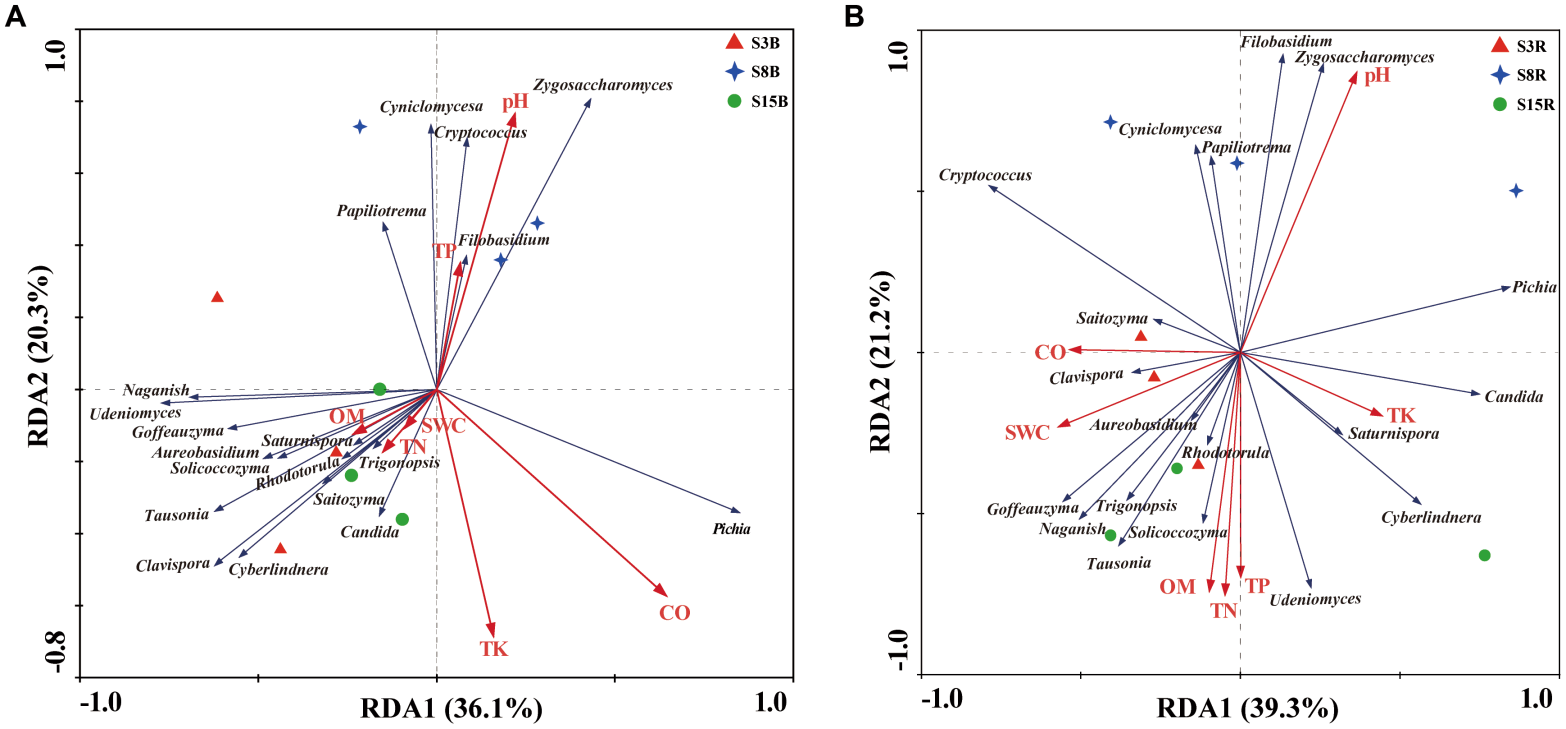
Redundancy analysis (RDA) of the correlation between the dominant yeast genera and soil physicochemical properties in **(A)** non-rhizosphere and **(B)** rhizosphere soil samples from peach trees of different ages. Red, blue, and green symbols in **(A)** and **(B)** represent non-rhizosphere and rhizosphere soil samples from 3-year-old (S3), 8-year-old (S8), and 15-year-old (S15) peach trees, respectively. Red and blue arrows represent the soil physical and chemical properties and genera, respectively. Soil physicochemical properties: pH, Conductivity (CO), Soil water content (SWC), Organic matter (OM), Total nitrogen (TN), Total phosphorus (TP), Total potassium (TK). Sample abbreviations are same as presented in Figure 1.

Moving on, redundancy analysis (RDA) based on yeast genera and soil physical and chemical properties in non-rhizosphere soil samples showed that the first and second RDA components explained 36.1% and 20.3% of the variation, respectively, for a total of 56.4% of the total variation (Figure 6A). The degree of influence of soil factors on yeast communities in non-rhizosphere soils was in the following order: CO (F-ratio: 2.06, *p* values: 0.04) > TK (F-ratio: 1.90, *p-*values: 0.086) > SWC (F-ratio: 1.18, *p-*values: 0.348) > pH (F-ratio: 1.08, *p-*values: 0.40) > TP (F-ratio: 1.08, *p-*values: 0.42) > TN (F-ratio: 0.33, *p-*values: 0.826) > OM (F-ratio: 1.29, *p-*values: 0.454). Among them, CO was significantly associated with the yeast communities in non-rhizosphere soils (*p* < 0.05). CO was positively correlated with *Pichia* but negatively correlated with *Cyniclomyces*, *Cryptococcus*, *Filobasidium*, and *Papiliotrema.*

Immediately after, we performed a RDA of the correlation between the yeast genera and soil physical and chemical properties in rhizosphere soil samples. The results show that the first and second RDA components explained 39.3% and 21.2% of the variation, respectively, for a total of 60.5% of the total variation (Figure 6B). The degree of influence of soil factors on yeast communities in rhizosphere soils was in the following order: pH (F-ratio: 1.97, *p-*values: 0.036) > TN (F-ratio: 1.54, *p-*values: 0.224) > CO (F-ratio: 1.27, *p-*values: 0.260) > TK (F-ratio: 1.70, *p-*values: 0.178) > SWC (F-ratio: 1.20, *p-*values: 0.344) > TP (F-ratio: 0.80, *p-*values: 0.548) > OM (F-ratio: 0.56, *p-*values: 0.650). pH was the soil factors that has significant effects on the distribution of yeast communities in rhizosphere soils (*p* < 0.05). pH was positively correlated with *Zygosaccharomyces*, *Filobasidium*, *Cyniclomyces,* and *Papiliotrema* but negatively correlated with *Clavispora*, *Trigonopsis*, *Tausonia*, *Solicoccozyma*, *Udeniomyces*, *Goffeauzyma*, and *Naganishia.*

The above results indicate there is a correlation between the yeast communities and soil physical and chemical properties, particularly CO and pH levels in the soil, and that soil chemical properties are important factors influencing the appearance of differences in yeast community structure in non-rhizosphere and rhizosphere soil samples.

### 3.5. Analysis of yeast co-occurrence networks and topological properties

**Figure 7:**
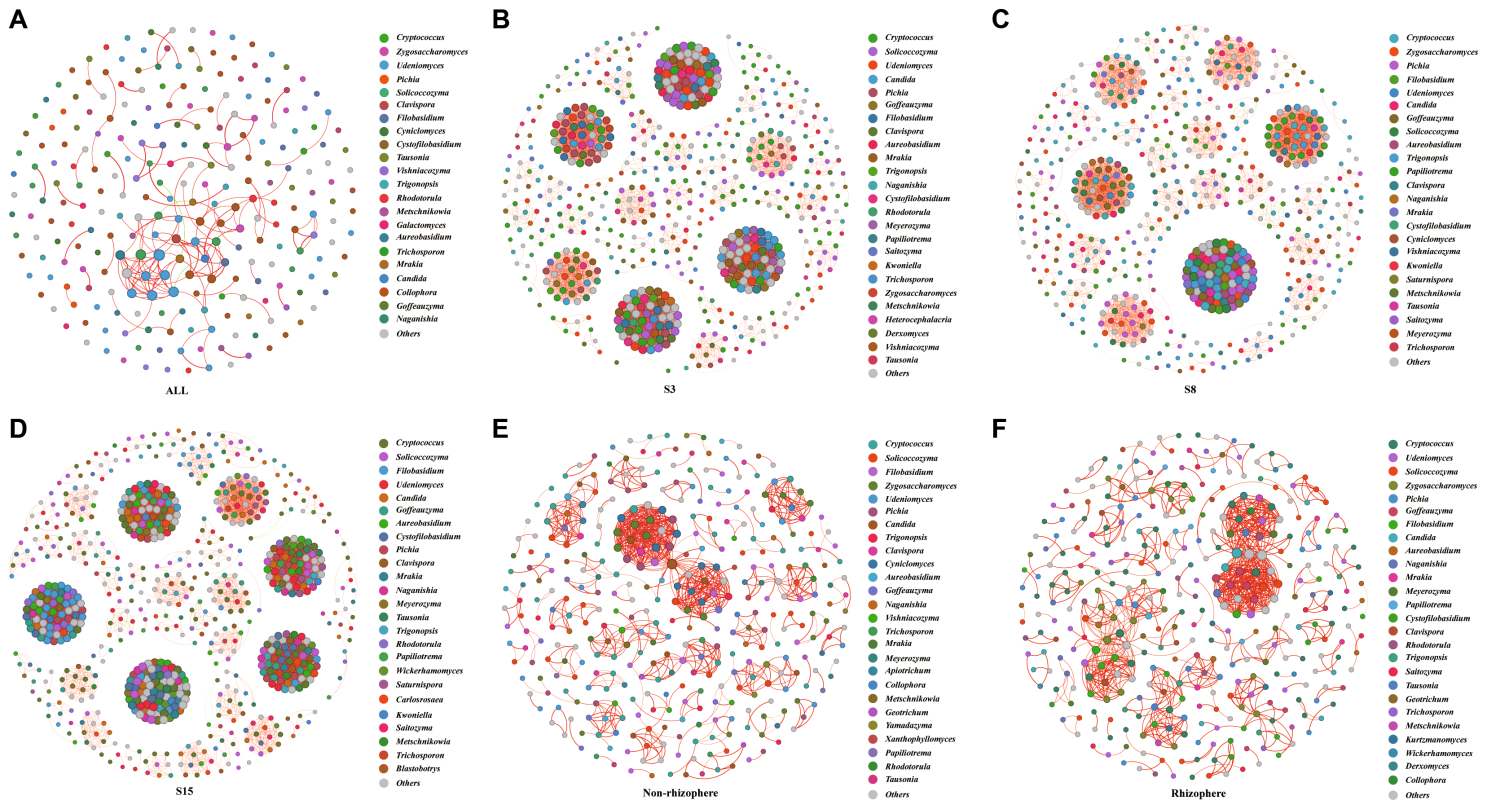
Network of co-occurring 90% cutoff OTUs based on correlation analysis. A connection stands for a strong (Spearman’s |r| > 0.6) and significant (*p*-value <0.01) correlation. Nodes in the network represent different genera (OTUs belonging to the same genera are grouped into the same color); the size of each node is proportional to the number of connections (that is, degree). A red edge represents a positive interaction, and a green edge represents a negative interaction. The thickness of the line is proportional to the correlation coefficient between OTUs. The greater the number of lines indicates the more closely related that OTU is to the others. Others indicated that yeast genera accounted for less than 1%. **(A)** ALL: All soil samples in this study. The remaining sample abbreviations **(B)** S3, **(C)** S8 **(D)** S15 **(E)** Non-rhizosphere **(F)** Rhizosphere are same as presented in Figures 1, 2.

To investigate the potential interactions of yeast communities and changes in co-occurrence networks at temporal and spatial scales, we constructed yeast co-occurrence networks based on random matrix theory in all samples, in samples of each tree age, and in non-rhizosphere and rhizosphere samples, respectively (Figure 7). The topological properties of the network indicate that the ALL co-occurrence network consists of 126 nodes and 153 edges with an average degree of 2.429, implying that each node is directly connected to approximately two other nodes. The average degree reveals the degree of connectivity of the components in the yeast network, and the higher the average degree, the higher the degree of network interactions. Secondly, the network average clustering coefficient (ACC), average path length (APL), and density of the ALL co-occurrence network are 0.644, 3.769, and 0.019, respectively. These three together reveal the tightness of each component of the network, where the smaller the APL is, the higher the network tightness. The modularity of the network was 0.809 (Values > 0.4 indicate that the network have modular structures), indicating a high degree of modularity and representing a high degree of classification of the community structure and function of yeast. Overall, complex relationships existed in the soil yeast community of the peach orchard (Table 6).

**Table 6:** Key topological features of yeast co-occurrence networks in each sample group.

Group	Nodes	Edges	Average degree	ACC	APL	Density	Positive edges	Negative edges	Modularity
ALL	126	153	2.429	0.644	3.769	0.019	150	3	0.809
S3	624	6,834	21.90	1	1	0.035	6,730	104	0.841
S8	599	6,595	22.02	1	1	0.037	6,495	100	0.754
S15	731	11,679	31.95	1	1	0.044	11,653	26	0.845
Non-rhizosphere	361	836	4.632	0.928	1.362	0.013	836	0	0.895
Rhizosphere	360	890	4.944	0.886	1.506	0.014	889	1	0.842

Sample abbreviations are as in Figures 1, 2. ACC, average clustering coefficient; and APL, average path length.

On the time scale, the degree of interactions and tightness of connections among components in the S15 yeast network were significantly higher than those in S3 and S8. In the case that the network average clustering coefficients (ACC) and the average path lengths (APL) of the three network graphs were consistent, the S15 network had the highest number of nodes (731) and edges (11679) and the highest average degree (31.95), density (0.44), and modularity (0.845). The next highest degree and tightness of network interactions was S3, and S8 was the lowest. At the spatial scale, the average degree (4.944), APL (1.506), and density (0.014) of the yeast co-occurrence network of rhizosphere soils were slightly higher, and the ACC (0.886) and modularity (0.842) were slightly lower than those of the non-rhizosphere, indicating a slightly higher degree of interactions and a slightly lower degree of tightness and modularity in the rhizosphere soil network compared to the non-rhizosphere soil. The number of positively correlated edges in the network was greater than the number of negatively correlated edges, both at the overall level and at the temporal and spatial scales, indicating greater synergy and less antagonism among yeast communities. The largest synergistic effect of yeast network was found in the S15 on the time scale, with more than 99.78% of the positive correlation edges, and in the rhizosphere network on the spatial scale, with 100% of the positive correlation edges.

The nodes in the yeast co-occurrence network were divided by genus level, and to explore the variation of core species in the peach orchard soil yeast co-occurrence network, we counted the degree of all genera in each network (Supplementary Tables S14–S19) and enumerated the top five ranked hub genera (Table 7). The analysis showed that nodes in the ALL, S3, S8, S15, non-rhizosphere, and rhizosphere networks belonged to 40, 74, 74, 82, 62, and 68 genera, respectively. Hub genera in the ALL yeast network were, in order, *Zygosaccharomyces* (39.87%), *Cryptococcus* (9.80%), *Pichia* (9.15%), *Udeniomyces* (6.54%), and *Clavispora* (5.88%). The composition and proportion of hub genera in each network changed with spatial and temporal changes. For example, *Pichia* accounted for 11.55% and 15.03% in the S3 and S8 networks, respectively, and only 6.92% in S15. The unique hub genera in the S3 and S15 networks were *Clavispora* and *Filobasidium*, respectively, and the unique hub genera in S8 were *Zygosaccharomyces* and *Trigonopsis*. The non-rhizosphere compared with the rhizosphere network each had two unique hub genera, *Pichia* and *Cyniclomyces* for the non-rhizosphere and *Goffeauzyma* and *Filobasidium* for the rhizosphere.

**Table 7:** The top five hub genera in co-occurrence networks for each sample group.

Sample groups	Genus				
ALL	*Zygosaccharomyces*	*Cryptococcus*	*Pichia*	*Udeniomyces*	*Clavispora*
S3	*Candida*	*Pichia*	*Clavispora*	*Cryptococcus*	*Aureobasidium*
S8	*Pichia*	*Zygosaccharomyces*	*Candida*	*Trigonopsis*	*Cryptococcus*
S15	*Candida*	*Filobasidium*	*Aureobasidium*	*Cryptococcus*	*Pichia*
Non-rhizosphere	*Zygosaccharomyces*	*Pichia*	*Candida*	*Cyniclomyces*	*Cryptococcus*
Rhizosphere	*Zygosaccharomyces*	*Cryptococcus*	*Candida*	*Goffeauzyma*	*Filobasidium*

Sample abbreviations are as in Figures 1, 2.

## 4. Discussion

### 4.1. Yeast diversity and community composition of peach orchard

Based on high-throughput sequencing technology we obtained a total of 3,103 yeast OTUs from peach orchard soil, identified as 114 genera belonging to Ascomycota and Basidiomycota (Tables 3, 4). The species richness and species diversity of the soil samples in this study (Table 2) were at a high level compared to other soil types (Yurkov et al., 2016; Vadkertiová et al., 2019). This suggests that peach orchard soils are rich in yeast resources, which is consistent with previous studies obtained using culturable methods (Wang et al., 2019). But compared to traditional culture methods, high-throughput sequencing technology is significantly more advantageous and can provide more comprehensive detection of species composition in habitats (Zebin et al., 2016). Also, the results of the Alpha diversity index among groups showed no significant differences in yeast community diversity between non-rhizosphere and rhizosphere soils at ages 3, 8, and 15 years (Table 2), which indicated that the overall yeast distribution in the peach orchard soil was relatively stable. It validates the conclusions obtained by previous studies that the composition of the soil microbial community under fruit trees is generally more stable than that of annual crops, as it is less likely to be disturbed by management practices (Vadkertiová et al., 2017; Mercado-Blanco et al., 2018).

In our study, Ascomycetous yeast genera were more numerous than Basidiomycetes yeast genera, which further validates the idea that Ascomycetous yeasts are usually more frequent and abundant in agricultural soils, orchards, and grasslands (Sláviková and Vadkertiová, 2003; Yurkov et al., 2012). In addition, we found that 33 yeast genera were detected in both non-rhizosphere and rhizosphere soils of 3-, 8-, and 15-year-old peach trees in the peach orchard, suggesting that these genera may be resident yeasts in peach orchard soils (Tables 3, 4). Among them, *Cryptococcus*, *Pichia*, *Candia*, *Rhodotorula*, and *Hanseniaspora* can be found in most soil types, but their diversity and abundance of these species vary from one habitat to another (Poliakova et al., 2001; Wang, 2007; Xu, 2009), this is supported by our findings. Apart from that, *Saitozyma*, *Solicoccozyma*, and *Goffeauzyma* are dominant yeasts in our study and are reported to be equally dominant in other soil types (França et al., 2016; Groenewald et al., 2018; Yurkov, 2018). In fact, not every yeast isolated from soil is a native soil dweller but may come from sources other than soil (Phaff et al., 1978; Phaff and Starmer, 1987). For example, some species of the Ascomycetous genera *Aureobasidium*, *Hanseniaspora*, *Metschnikowia*, *Saccharomyces*, and *Pichia*, as well as the Basidiomycetes genera *Rhodotorula*, *Cystobasidium*, *Vishniacozyma*, and *Sporobolomyces* that were detected in this study, are usually dominant species isolated from the above-ground vegetative organs (leaves, flowers, and fruits) of the plant (SlÁviková et al., 2009; Sipiczki, 2016; Yurkov, 2018). This may be due to the fact that our sampling time was at the peak of the peach tree’s fruiting season, and there would be epiphytic yeast entering the soil with the fallen peaches or leaves. Furthermore, the rare yeast genera discovered in this study accounted for approximately 13.16% of the yeast genera in all soil samples (Tables 3, 4), which is significantly lower than the proportion of rare yeasts found in other orchards (Vadkertiová et al., 2019), forest (França et al., 2016), grassland, and shrub soils (Yurkov et al., 2016). Previous studies have shown that reduced precipitation leads to increased populations of rare species in soil habitats (Yurkov et al., 2016). In contrast, the field management pattern of the peach orchard in this study provided sufficient water, so this may be more favorable for yeast survival.

### 4.2. Spatial and temporal characteristics of soil yeast communities In peach orchard

Although there were no significant differences in yeast diversity among the groups, their community structure showed significant variation at temporal and spatial scales, particularly at the temporal scale (Figure 5). The yeast community was most evenly distributed at 15 years compared to the soil yeast community composition at 3 and 8 years (Figure 3; Tables 3, 4). This indicates that the soil yeast community was already more stable at 15 years. Previous studies have shown that an increase in shared species diversity can improve the stability of microbial communities (Wang et al., 2013). The variation in the number of shared yeast OTUs among the three ages in this study also proved this (Figure 2A). And, the stability of the yeast community also contributes to the resistance of peach trees. In addition, the abundance of the shared dominant genera *Zygosaccharomyces* and *Aureobasidium* increased significantly in 8- and 15-year-old peach soils, respectively. *Zygosaccharomyces* has been reported to be involved in the solubilization of soil insoluble phosphate, which may be related to the high phosphorus demand of 8-year-old peach trees (Gizaw et al., 2017; Petkova et al., 2022). Moreover, 8-year-old peach trees may accumulate pathogenic fungi with increasing age, and *Zygosaccharomyces* also has the ability to produce siderophore compounds (iron (III) ion compounds) that inhibit the growth of fungal phytopathogens (Hider and Kong, 2010; Petkova et al., 2022). *Aureobasidium* has been shown to be effective against postharvest fruit pathogens (Di Francesco et al., 2020; Podgórska-Kryszczuk, 2023).

In terms of spatial scale, we found that the total number of yeast OTUs in peach rhizosphere soil was higher than that in non-rhizosphere soil. In agreement with a previous study, rhizosphere microorganisms have better abundance and diversity than non-rhizosphere microorganisms (Yue et al., 2018). In addition, the number of shared OTUs in non-rhizosphere soil samples among different tree ages was higher than the number of shared OTUs in their rhizosphere samples (Figure 2). This indicates that the yeast community is more specific in the rhizosphere soil of different tree ages, possibly influenced by factors such as the rhizosphere secretion of peach trees. It has been reported that root secretions have a selective role in shaping the rhizosphere microbial community structure, which is unique of different plants (Paterson et al., 2007). For example, *Candida*, *Geotrichum*, *Rhodotorula*, and *Meyerozyma* were all detected in all rhizosphere samples in this study, and their representative species are thought to be associated with nitrification in the soil, where nitrite and phosphate are solubilized *in vitro* to nitrate (Chen et al., 2012; Nakayan et al., 2013). It is worth noting that the abundance of the yeast genera *Metschnikowia*, *Wickerhamomyces*, *Geotrichum*, and *Torulaspora* was significantly increased in the 15-year peach rhizosphere soil samples compared to the 3- and 8-year samples. The results of the present study suggest that the increase in abundance of the first three may be caused by the accumulation of a large number of pathogens due to the increase in the number of years of colonization of peach trees. Some representative species of *Metschnikowia* (Sipiczki, 2020; Wang et al., 2021), *Wickerhamomyces* (Lanhuang et al., 2022), and *Geotrichum* (Kawtharani et al., 2022) have been shown to be antagonistic to various pathogens and can be widely used as biocontrol agents in organic agriculture. *T. delbrueckii* in the genus *Torulaspora* has been reported to be able to produce phytase under certain conditions, increasing the nutritional content of peach and improving the absorption of trace elements in peach by humans (Kaur et al., 2007). All these results indicate that the rhizosphere microbial community and plant growth and development are mutually influential. In summary, the changes in yeast community structure in the soil of the peach orchard in this study were consistent with previous findings that soil microbial diversity has certain spatial and temporal characteristics (Kowalchuk et al., 2002; Yue et al., 2018).

### 4.3. The relationship between yeast community structure and soil factors in peach orchard

The drivers of yeast community assembly in soils are more complex and mainly include the effects of environmental conditions and vegetation (Mašínová et al., 2017), among which soil physicochemical properties are the key factors. Because the soil physical and chemical properties reflect both the growth state of plants and the survival conditions of yeast in the soil. In this study, there were significant differences in soil physicochemical properties among the samples, especially in pH, organic matter (OM), total nitrogen (TN), and total phosphorus (TP; Table 5). The results of the RDA analysis clearly revealed that conductivity (CO) and pH were the main factors influencing the structure of the yeast community (Figure 6). The same conclusion was reached in a previous study of the diversity of soil yeasts isolated from South Victoria Land, Antarctica (Connell et al., 2008). The soil conductivity CO reflects the amount of salt in the soil water solution. Generally speaking, the higher the CO value of the soil within a suitable range, the more fast-acting nutrients are available to the plant (Zhang et al., 2009). We found a significant positive correlation between *pichia* and CO, suggesting that *pichia* may be beneficial to plant growth. In addition, soil pH is also one of the main factors influencing the composition of the soil yeast community. It has been described as the “master soil variable” that influences a myriad of soil biological, chemical, and physical properties and processes and affects plant growth and biomass production (Minasny et al., 2016). The pH of the soil samples collected for this study ranged from 7.76 to 8.41, with an overall weak alkalinity. We found a negative correlation with pH for most of the yeast genera, indicating that overall the yeast community still prefers an acidic environment, which is a common characteristic of yeasts (Chen, 2012). In contrast, the genera *Zygosaccharomyces*, *Filobasidium*, *Cyniclomyces*, and *Papiliotrema* in this study showed a positive correlation with pH, indicating that these three yeast genera prefer alkaline environments for survival.

In this study, we found that 15-year-old peach inter-root soils had the lowest pH but significantly higher levels of OM, TN, and TP than the other samples (*p* < 0.05; Table 5). This indicates that the 15-year rhizosphere soil fertility was higher. Because pH can affect soil function and plant nutrition effectiveness by influencing the chemical solubility and availability of essential plant nutrients, pesticide performance, and organic matter decomposition (McCauley et al., 2009). Furthermore, *Tausonia*, *Solicoccozyma*, *Trigonopsis*, and *Goffeauzyma* in this study showed positive correlations with OM, TN, and TP, indicating that they grow in abundance in nutrient-rich environments and can be used to indicate soil fertility. In summary, soil CO and pH play an important role in coordinating crop growth and soil yeast community structure. Tracing the factors that contribute to differences in the structure of soil yeast communities helps us better provide solutions to improve soil ecology and thus contribute to the sustainable development of fruit trees.

### 4.4. Co-occurrence patterns of soil yeasts in peach orchard

To further understand the survival mechanisms of yeast communities in peach orchard soils, we conducted a co-occurrence network analysis of yeast communities in soils in multiple dimensions: overall, temporal, and spatial (Figure 7). Co-occurrence network analysis has now been widely used in the field of microbiology. It measures the interactions between different microbial taxa by correlating the abundance of microbial taxa across multiple soil samples and extracting simple patterns from complex interactions to identify cooperative or competitive relationships between species and further infer community assembly and evolutionary mechanisms (Goberna and Verdú, 2022; Guseva et al., 2022). By calculating the degree of the network nodes, the central node microorganisms of the network can be screened for microorganisms with potential ecological functions (Shetty et al., 2017). The results of this study show that there is significant spatial and temporal specificity in soil yeast community interactions in the peach orchard, with particularly pronounced variation on the temporal scale. This is consistent with the changes in yeast community structure. The degree of interactions and connection tightness of the 15-year-old yeast community were higher than those of the 3-year-old and 8-year-old ones, while there were obvious yeast network modularity, core yeast genera, and network connection nodes, indicating the reliability of the interactions among the 15-year-old soil yeasts. This indicates that the yeast community has acquired certain structural and functional stability in its long-term evolution with increasing age. In addition, there were more positive connections than negative connections in the peach orchard soil yeast network, indicating that yeasts in peach orchard soil prefer to coexist in a synergistic mutualistic manner, and the strongest synergistic effect among the three tree-aged soil yeast networks was found among 15-year-old soil yeasts, which further supports the strong stability of their network structure and function.

Comparatively, although the rhizosphere network was more interactions than the non-rhizosphere, the degree of connectivity tightness and modularity were lower than the non-rhizosphere. It indicates that the interactions between rhizosphere soil yeasts are more random and do not have stability and reliability. And the fact that yeast synergistic effect is lower in the rhizosphere network than in the non-rhizosphere network also illustrates the same issue. Unlike the previous conclusions obtained that a more stable microbial network exists in rhizosphere soils compared to non-rhizosphere soils of wheat (Fan et al., 2018). This may be due to the fact that yeast is more sensitive to environmental changes in soil only as a taxon of fungi, while yeast in inter-rhizosphere soil is more susceptible to the influence of plant roots compared to non-rhizosphere yeast, in addition to the influence of the environment (Paterson et al., 2007; Fan et al., 2018).

At the same time, our observation of each network graph revealed that OTU nodes in low-abundance yeast genera are also likely to have a high degree (Figure 7), which reaffirms the important role of low-abundance microbial genera in maintaining the stability and function of microbial communities (Guo et al., 2022). Furthermore, we found that each network differed in the composition of the hub yeast genera, which may also be a response of the peach orchard soil yeast community to temporal and spatial changes. Hubs in the network are usually defined as keystone species because if these taxa are removed, the network may also split; thus, they play a crucial role in the network structure and can be identified as targets for microbial regulation to improve crop productivity (Olesen et al., 2007). In this study, we can take the hub yeast genera in the network as a reference and then use its related soil factor as a condition for the improvement of peach orchard soil to improve the quality of soil microbiology and finally achieve the purpose of maintaining the health of peach trees and promoting the quality growth of peach trees.

## 5. Conclusion

In this study, we found for the first time the living strategies of soil yeasts at the spatial and temporal scales of perennial peach trees. Unlike soil yeast diversity in the peach orchard, yeast community structure varies significantly on spatial and temporal scales. Soil factors such as CO and pH were the main factors influencing the differences in yeast community structure. This study reveals the changes in the diversity and community structure of non-rhizosphere and rhizosphere soil yeast at different ages in the peach orchard and the factors affecting them, as well as the spatio-temporal response of the soil yeast network in peach orchards, providing new insights into the role of soil yeast resources in achieving sustainable agricultural development in peach orchards and its spatio-temporal adaptation mechanisms.

## Data availability statement

The data of high throughput sequencing in this project has been deposited in the Sequence Read Archive (SRA) of the National Center for Biotechnology Information (NCBI) under the accession number PRJNA992790 (https://www.ncbi.nlm.nih.gov/sra/?term=PRJNA992790).

## Ethics statement

Ethical approval is not applicable in the case of the study. However, the collection of the soil samples from the peach orchard was verbally permitted by the farm owner.

## Author contributions

SSZ designed and performed the experiments, analyzed the data, and drafted the manuscript. YLC helped design experiments, analyzed the data, and drafted the manuscript. YL performed sample collection, DNA extraction, PCR amplification and analyzed part of the data. JX performed sample collection and soil chemical property analysis. YHL performed sample collection, DNA extraction, and PCR amplification. YFS designed and performed the experiments and analyzed data. All authors contributed to the article and approved the submitted version.

## Funding

The work was funded by the National Natural Science Foundation of China (project number: 31860003).

## Conflict of interest

The authors declare that the research was conducted in the absence of any commercial or financial relationships that could be construed as a potential conflict of interest.

## Publisher’s note

All claims expressed in this article are solely those of the authors and do not necessarily represent those of their affiliated organizations, or those of the publisher, the editors and the reviewers. Any product that may be evaluated in this article, or claim that may be made by its manufacturer, is not guaranteed or endorsed by the publisher.

## Footnotes

^1^https://www.cpc.ncep.noaa.gov/

^2^https://disc.gsfc.nasa.gov/

## Abbreviations

OTU: Operational taxonomic units
CO: Conductivity
SWC: Soil water content
OM: Organic matter
TN: Total nitrogen
TP: Total phosphorus
TK: Total potassium
PRECTP: Average annual precipitation
TEMP: Average annual temperature
LST: Average annual land surface temperature
RH: Average annual relative humidity
EVLAND: Average annual evaporation land
PCoA: Principal coordinates analysis
RDA: Redundancy analysis
