# Supplementary material for: Effects of temporal and spatial scales on soil yeast communities in the peach orchard
Source: Front Microbiol. 2023 Sep 19;14:1226142. doi: 10.3389/fmicb.2023.1226142 (PMC10546340; doi:10.3389/fmicb.2023.1226142)
Supplement: Supplementary file 1 [file Data_Sheet_1.ZIP › Table S1.docx]

**Table S1.** **DNA concentration and purity of 18 peach orchard soil samples**

| **Sample**  **name** | **DNA concentration**  **(ng/µl)** | **DNA purity** | | **Volume** **(µl)** | **Total amount (μg)** |
| --- | --- | --- | --- | --- | --- |
|  |  | **OD260/280** | **OD260/230** |  |  |
| S3B1 | 46.20 | 1.82 | 1.15 | 50 | 2.31 |
| S3B2 | 51.80 | 1.87 | 1.24 | 50 | 2.59 |
| S3B3 | 22.00 | 1.90 | 0.89 | 50 | 1.10 |
| S3R1 | 17.60 | 1.91 | 0.67 | 50 | 0.88 |
| S3R2 | 27.40 | 1.86 | 0.96 | 50 | 1.37 |
| S3R3 | 30.20 | 1.60 | 0.79 | 50 | 1.51 |
| S8B1 | 34.80 | 1.89 | 1.19 | 50 | 1.74 |
| S8B2 | 20.40 | 1.87 | 0.75 | 50 | 1.02 |
| S8B3 | 20.80 | 1.96 | 0.97 | 50 | 1.04 |
| S8R1 | 16.60 | 1.87 | 0.83 | 50 | 0.83 |
| S8R2 | 18.60 | 1.90 | 0.87 | 50 | 0.93 |
| S8R3 | 19.00 | 1.91 | 0.57 | 50 | 0.95 |
| S15B1 | 20.80 | 1.92 | 0.96 | 50 | 1.04 |
| S15B2 | 16.20 | 2.12 | 0.17 | 50 | 0.81 |
| S15B3 | 25.40 | 1.89 | 1.06 | 50 | 1.27 |
| S15R1 | 29.40 | 1.87 | 1.03 | 50 | 1.47 |
| S15R2 | 23.80 | 1.89 | 0.97 | 50 | 1.19 |
| S15R3 | 28.20 | 1.80 | 1.00 | 50 | 1.41 |
